# Optical coherence tomography angiography as a novel approach to contactless evaluation of sublingual microcirculation: A proof of principle study

**DOI:** 10.1038/s41598-020-62128-2

**Published:** 2020-03-25

**Authors:** Michael Hessler, Pieter Nelis, Christian Ertmer, Maged Alnawaiseh, Florian Lehmann, Christina Schmidt, Tim-Gerald Kampmeier, Sebastian Willy Rehberg, Philip-Helge Arnemann, Alexandros Rovas

**Affiliations:** 1 Department of Anesthesiology, Intensive Care, and Pain Therapy, University Hospital Muenster, Albert-Schweitzer-Campus 1, Building A1, Muenster, Germany; 2 Department of Ophthalmology, University Hospital Muenster, Domagkstraße 15, Muenster, Germany; 3 Department of Ophthalmology, University of Brussels (VUB), Laarbeeklaan 101, Jette, Belgium; 4 Department of Anesthesiology, Intensive Care, Emergency Medicine, Transfusion Medicine and Pain Therapy, Protestant Hospital of the Bethel Foundation, Burgsteig, Bielefeld, Germany; 5 Department of Medicine D, Division of General Internal Medicine, Nephrology, and Rheumatology, University Hospital Muenster, Albert-Schweitzer-Campus 1, Muenster, Germany

**Keywords:** Diagnosis, Diagnosis, Three-dimensional imaging, Three-dimensional imaging, Translational research

## Abstract

Microcirculatory disorders are crucial in pathophysiology of organ dysfunction in critical illness. Evaluation of sublingual microcirculation is not routinely conducted in daily practice due to time-consuming analysis and susceptibility to artifacts. We investigated the suitability of optical coherence tomography angiography (OCTA) for contactless evaluation of sublingual microcirculation. Sublingual microcirculation was imaged in 10 healthy volunteers, using an OCTA device and an incident dark field (IDF) illumination microscopy (current gold standard). OCTA images were analyzed with regard to flow density and perfused vessel density (PVD_**byOCTA**_). IDF videos were analyzed following current recommendations. Flow density was automatically extracted from OCTA images (whole en face 48.9% [43.2; 54.5]; central ring 52.6% [43.6; 60.6]). PVD_**byOCTA**_ did not differ from the PVD calculated from IDF videos (PVD_**byOCTA**_ 18.6 mm/mm² [18.0; 21.7]) vs. PVD_**byIDF**_ 21.0 mm/mm² [17.5; 22.9]; p = 0.430). Analysis according to Bland-Altman revealed a mean bias of 0.95 mm/mm² (95% Confidence interval −1.34 to 3.25) between PVD_**byOCTA**_ and **PVD**_**byIDF**_ with limits of agreement of −5.34 to 7.24 mm/mm². This study is the first to demonstrate the suitability of OCTA for evaluating sublingual microcirculation. Comparison of the perfused vessel density between methods showed a plausible level of agreement.

## Introduction

In recent years, research has highlighted the importance of the microcirculation (vessels smaller than 100 μm) in the pathophysiology of diseases and organ dysfunction in critical illness. It is known that blood flow in the microcirculation is often impaired in critically ill patients and altered blood flow in the microcirculation is associated with outcome^1–6^. A decoupling of macro- and microcirculation (“*loss of hemodynamic coherence*”), as can occur, for example, in advanced stages of septic shock is of particular interest here^7^. In such conditions, macrohemodynamic parameters such as cardiac output and perfusion pressure are no longer indicative of perfusion in the microcirculation. The aim of hemodynamic therapy should therefore be restoration not only of the macrocirculation but also the microcirculation^7^. Hence the demand for bedside methods to monitor the microcirculation.

Bedside analysis of the microcirculation became possible with the introduction of modern handheld video microscopes using sidestream dark field (SDF) imaging or incident dark field (IDF) illumination technology^8–10^. Unfortunately, the analysis of the microcirculation has not yet become established in routine clinical practice, as video microscopy of capillary blood flow has so far been limited by artifacts (e.g., pressure artifacts at the necessary point of contact with the tissue) and time-consuming semi-manual evaluation^11,12^. However, information about microcirculatory perfusion would be of great interest in intensive care to predict outcome and potentially guide therapy^13,14^.

Optical coherence tomography angiography (OCTA) is relatively new and has so far been used mainly for high-resolution imaging of the retina and choroid vascularization. With OCTA, the blood flow in the microcirculation of retina and choroid can be evaluated non-invasively, reproducibly and with an automatic analysis at the bedside. As a result, the use of OCTA in ophthalmic research and clinical practice has become widespread in recent years^15–23^. Recent experimental studies have demonstrated the potential usefulness of OCTA for evaluating microcirculatory alternations in critical illness by investigating the microcirculatory changes in the retina during hemorrhagic shock, septic shock and resuscitation^24,25^.

The sublingual area is the most frequently used site for evaluating the microcirculation in critically ill patients^26^. However, no study has yet investigated the suitability of OCTA for evaluation of the sublingual microcirculation. The aim of the present pilot study was therefore to assess the feasibility of using OCTA for contactless evaluation of the sublingual microcirculation in healthy volunteers and to compare this new approach with an established method for monitoring sublingual microcirculation.

## Results

### Study participants

Ten healthy participants were recruited for the study from our research department. Median age was 30 [27; 32] years. The participants had had no previous illnesses and were not on any medication. Further demographic data are shown in Table 1.

**Table 1:** Characteristics of the study participants.

Variable (unit)		Median [interquartile range]
N		10
Sex (No.)	male	5
	female	5
Body weight (kg)		75 [60; 79]
Body height (cm)		174 [167; 181]
Body mass index (kg·m^−2^)		23.5 [20.1; 25.6]
SAP (mmHg)		130 [116; 139]
DAP (mmHg)		80 [73; 89]
MAP (mmHg)		96 [90; 104]
HR (beats per min)		82 [77; 85]
S_p_O_2_ (%)		99 [98; 99]

DAP, diastolic arterial pressure; HR, heart rate; MAP, mean arterial pressure; SAP, systolic arterial pressure; SpO2, peripheral oxygen saturation.

### Imaging of sublingual microcirculation with OCTA

**Figure 1:**
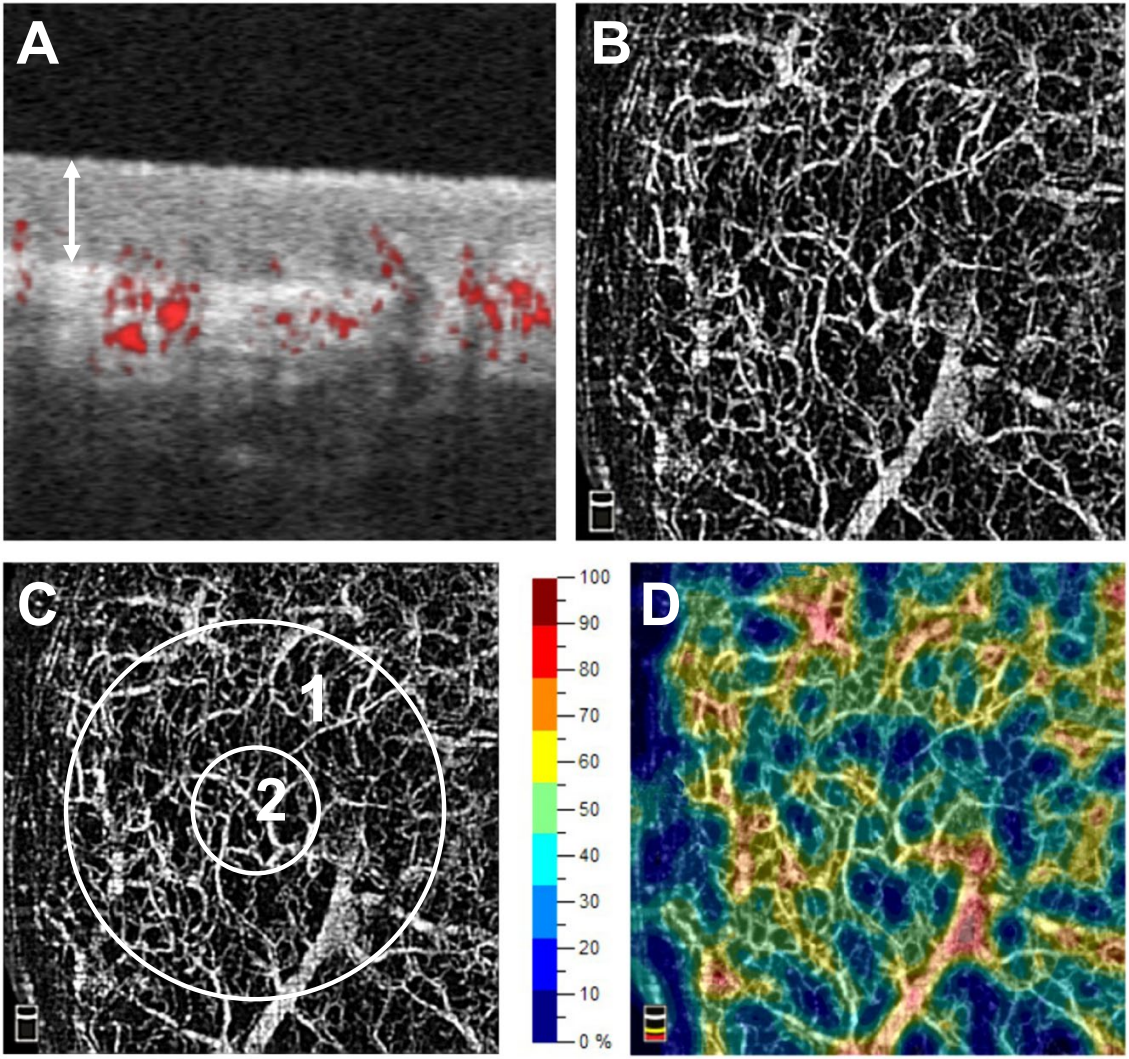
Optical coherence tomography angiograms of the sublingual microcirculation. Optical coherence tomography (OCT) angiograms of the sublingual microcirculation. Cross-sectional image (B-scan; (**A**) with perfused vessels visible as red dots. The white double arrow shows the stratified squamous epithelium. En face OCT angiograms (**B**,**C**) and color-coded OCT angiogram (**D**) of the same area of the sublingual mucosa. Circle 2 (**C**) indicates the region which was used for calculation of the flow density (central ring). The flow density (whole en face) is the average flow density of circles 1 and 2. A: 1 × 1 mm; B – D: 3 × 3 mm scans.

**Figure 2:**
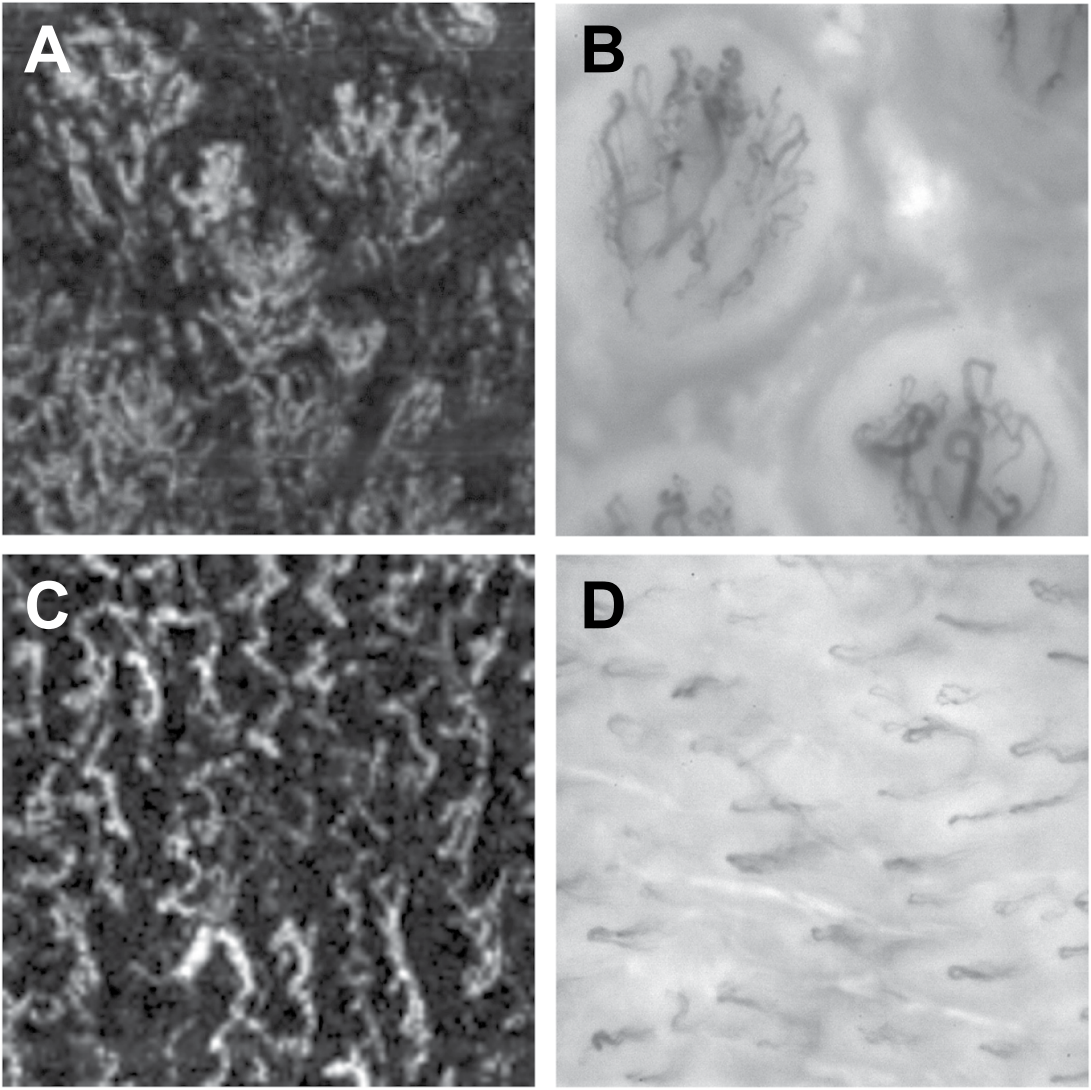
Comparison of characteristic vessel configurations of the oral mucosa imaged by optical coherence tomography angiography and incident dark field illumination. Vessels of the tongue papillae recorded by optical coherence tomography angiography (OCTA; (**A**), 0.75 × 0.75 mm) and incident dark field illumination (IDF; (**B**), 1.1 × 1.1 mm). Vascular loops of the sublingual microcirculation recorded by OCTA (**C**), 1.1 × 1.1 mm) and IDF (**D**), 1.1 × 1.1 mm).

With OCTA it was possible to obtain detailed images of the sublingual microcirculation. In longitudinal sections of the sublingual mucosa (Fig. 1A), the stratified squamous epithelium could be distinguished from the lamina propria. The median thickness of the stratified squamous epithelium was 120 μm [104; 149]. In these images, vessels could be identified by color coding as red dots (cross-cut) or as red lines (cut longitudinally). The vascular leading layer, which could be detected by OCTA had a median thickness of 409 μm [361; 451]. Most vessels were detected in the lamina propria and interdigitated with the rete ridges of the overlying epithelium. Single capillaries followed the connective tissue papillae of the lamina propria and reached up to half the thickness of the stratified squamous epithelium. Individual capillaries as well as larger, deeper vessels could be recognized in transversal images (*en face* images, Fig. 1,B–D) of the sublingual microcirculation. In addition, characteristic vessel formations of the oral microcirculation could be identified by OCTA (Fig. 2).

Compared to OCTA images, those produced by IDF were sharper so that individual vessels were more easily distinguishable (Fig. 2, B,D). In contrast, capillaries located deeper in the tissue were revealed with OCTA, which did not show up on IDF illumination imaging (for example, vascular connections between vessel loops; Fig. 2,C).

### Quantification of sublingual flow density and perfused vessel density

**Figure 3:**
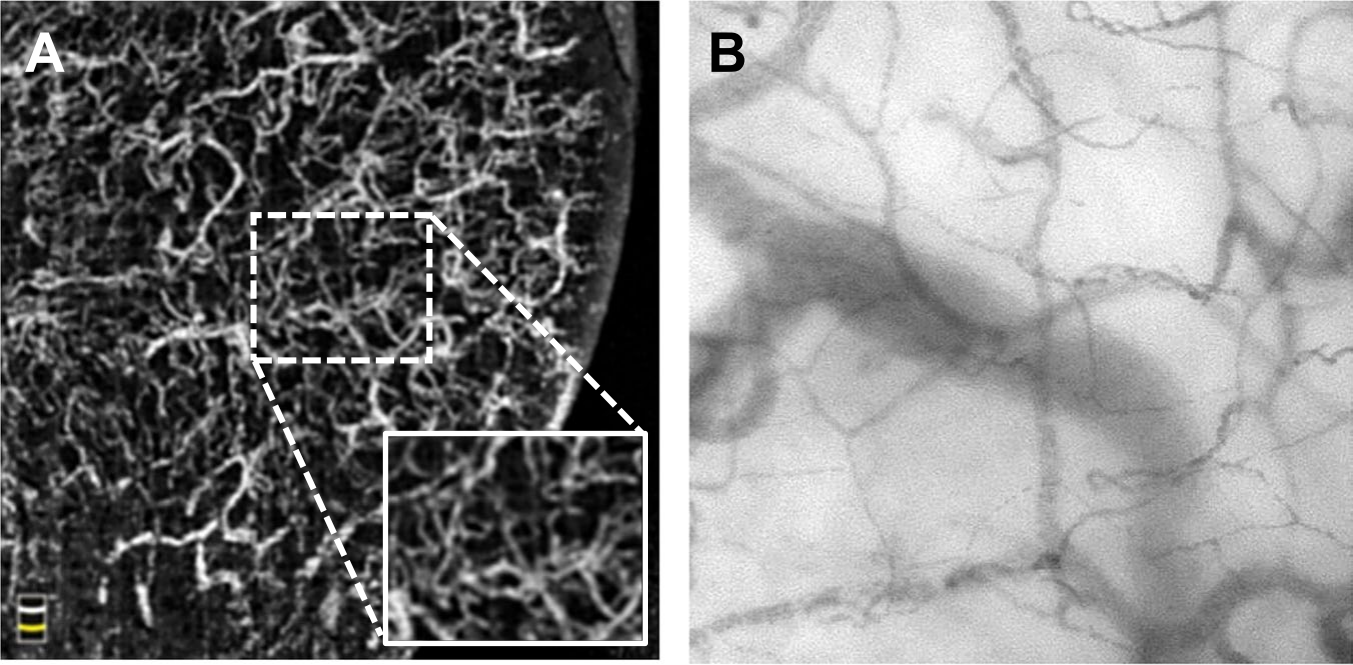
Analysis of the perfused vessel density in optical coherence tomography angiograms of the sublingual microcirculation. En face optical coherence tomography (OCT) angiogram (**A**) of the sublingual microcirculation. For analysis of the perfused vessel density (PVD_by OCTA_), a picture section (box with dashed line; 858 × 688 µm) was exported from the OCT angiogram, within which the vessel length relative to the image size was determined. Picture (**B**) shows a picture of the sublingual microcirculation recorded by incident dark field illumination (688 × 688 µm).

**Figure 4:**
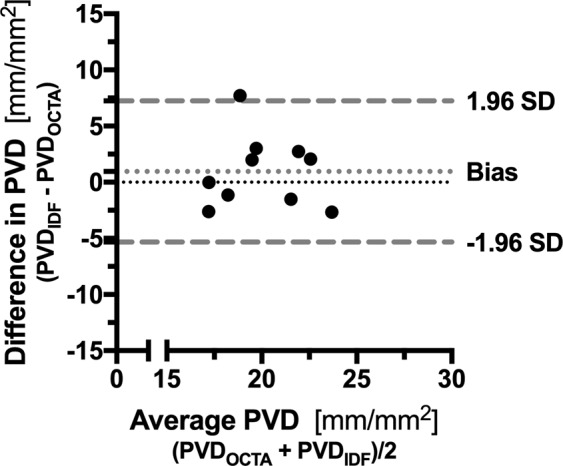
Bland-Altman plot for the perfused vessel density by incident dark field illumination and optical coherence tomography angiography (n = 10). Dotted, light gray line represents the mean difference whereas upper and lower dashed, light gray lines represent the limits of agreement (equivalent to ± 1.96 standard deviation of mean difference). Abbreviations: IDF, incident dark field illumination, OCTA, optical coherence tomography angiography; PVD, perfused vessel density; SD, standard deviation.

The Flow density_WF_ and the Flow density_Central_ were automatically calculated in the OCTA images of the sublingual microcirculation (Fig. 1,C and Table 2). The IDF illumination videos of the sublingual microcirculation used for the analysis yielded sufficient image quality (MIQS 1.4 [1.3; 2.4]). Table 2 shows the results of the manual analysis of the IDF videos. The median perfused vessel density (PVD) PVD_by OCTA_ of the sublingual microcirculation (Fig. 3) was similar to the PVD_by IDF_ (p = 0.430; Table 2 and Additional File 1, Figure A3). Analysis according to Bland and Altman^27^ revealed a mean bias of 0.95 mm/mm² (95% Confidence interval −1.34 to 3.24) between PVD_by OCTA_ and PVD_by IDF_ with Limits of Agreement (LOA) of −5.33 to 7.24 mm/mm² and no evidence of proportional bias. Figure 4 presents the Bland-Altman plot for PVD_by OCTA_ and PVD_by IDF_. Spearman’s rank correlation coefficients (ρ) between the automatically calculated Flow density_WF_ in the superficial retinal OCT angiogram and the manually measured PVD_by OCTA_ or PVD_by IDF_ were as follows: PVD_by OCTA_ ρ = 0.600, p = 0.067 and PVD_by IDF_ ρ = −0.091, p = 0.803. For the relationship between the Flow density_Central_ and PVD_by OCTA_ or PVD_by IDF_ the following Spearman’s rank correlation coefficients were calculated: PVD_by OCTA_ ρ = 0.588, p = 0.074 and PVD_by IDF_ ρ = −0.200, p = 0.580.

**Table 2:** Results of sublingual microcirculation measured by incident dark field illumination and optical coherence tomography angiography.

Parameter (unit)	Median [interquartile range]
*IDF Illumination of sublingual microcirculation*	
TVD (mm ∙ mm^−2^)	21.9 [17.6; 23.4]
PVD_by IDF_ (mm ∙ mm^−2^)	21.0 [17.5; 22.9]
PPV (%)	97.8 [96.3; 99.8]
MFI_by quadrants_	2.9 [2.9; 3.0]
*OCTA of sublingual microcirculation*	
Flow density_WF_ (%)	48.9 [43.2; 54.5]
Flow density_Central_ (%)	52.6 [43.6; 60.6]
PVD_by OCTA_ (mm ∙ mm^−2^)	18.6 [18.0; 21.7]

MFI_by quadrants_, microvascular flow index; OCTA, optical coherence tomography angiography; PPV, proportion of perfused vessel; PVD_by IDF_, perfused vessel density in IDF illumination videos; PVD_by OCTA_, perfused vessel density in OCTA images, TVD, total vessel density.

## Discussion

This study is the first to demonstrate the suitability of OCTA for contactless evaluation of the sublingual microcirculation in healthy volunteers. It was possible to identify in detail characteristic vessel configurations and the layered structure of the sublingual microcirculation. Comparison of the PVD based on OCTA images and IDF illumination videos of the sublingual microcirculation, as a surrogate for the diffusive capacity, showed a plausible level of agreement.

As a contactless and non-invasive procedure, OCTA has recently found clinical application, especially in ophthalmology, since it allows generation of high-resolution images of the perfusion of the anterior eye segment, choroid and retina. Several experimental and clinical studies have shown the reliability and validity of OCTA for monitoring microvascular perfusion of the eye^28–31^. In the present study, OCTA was used successfully to evaluate the sublingual microcirculation in detail. The (histological) layered structure of the sublingual mucosa could be reconstructed in OCTA B-scans. Among other things, the measured thickness of the sublingual stratified squamous epithelium corresponded to published values^32,33^. Due to a lack of concrete anatomical landmarks in the sublingual region (such as the macula or the entry of the optic nerve into the fundus for the eye), no direct comparison between IDF Illumination videos and OCTA images of exactly the same sublingual region was possible in the current study. However, it was possible to identify characteristic vessel configurations of the oral and sublingual mucosa, which were known from IDF and SDF videos of the microcirculation (Fig. 2).

Video microscopy using SDF and IDF is the current gold standard for evaluating the sublingual microcirculation^26^. In this study, videos of the microcirculation were recorded using IDF illumination imaging, which allowed delineation of individual erythrocytes in capillaries (Fig. 3,B). Imaging by IDF and SDF is based on the absorption of green light by hemoglobin, while unabsorbed light is reflected by tissue, forming a bright background. In contrast, vessel detection by OCTA is based on the assessment of signal changes of light, emitted by the OCT device and reflected by tissue, through blood flow. This explains why no individual erythrocytes can be differentiated in vessels by OCTA and why the vessel borders are slightly blurred (Fig. 3,A). In this context, it is important to note that vessels delineated in OCTA images did not correspond with vessels in a still image of an IDF-generated video of the microcirculation. In contrast to a video still image, an OCTA image shows whether or not there is blood flow. As stated above, this allows automatic calculation of vessel density in a defined region.

In the current study, it was possible to determine perfused vessel density in the OCTA images of the sublingual microcirculation which agreed broadly with that obtained from the IDF videos. The analysis according to Bland-Altman (Fig. 4) may be interpreted as a plausible agreement between the two measurement methods, which may be due to the similar principles by which the perfused vessel density was determined. The PVD_by IDF_ was calculated from the proportion of vessel from the TVD, which were perfused at least sluggishly or continuously. For the determination of the PVD_by OCTA_, as described above, a blood flow must be detected by the OCTA device, resulting in a vessel being delineated in the OCTA image. Although the PVD_by IDF_ and PVD_by OCTA_ did not differ statistically, the Bland-Altman plot tends to show higher values for PVD_by IDF_ and LOA may be interpreted as relatively large (Fig. 4). Various factors may account for this observation: First, the vascular network for determining the PVD_by OCTA_ was based on cross-sectional images (B-scans) of the sublingual microcirculation. The segmentation borders for vascular detection may therefore have been set wrong. Another reason could be that blood flow velocity in vessels had to exceed a certain threshold before the blood flow was detected. However, this threshold is approximately <0.3 mm/s in modern OCTA devices^34^, while the mean capillary blood flow velocity in healthy subjects is approximately 1.3 mm/s^4^. Finally, the resolution of the OCTA device used in the current study is about 5–15 µm depending on the orientation (axial or transversal), so that small capillaries may not have been detected. In this context, OCTA devices with a transversal resolution of less than 5 µm have recently been developed^35^. In summary, this pilot study showed plausible agreement between IDF and OCTA-derived measures of PVD, but the novel approach to use OCTA to evaluate the sublingual microcirculation needs further improvement and validation before comparability between the two methods can be assumed.

This study was not able to find a significant correlation between the automatically calculated flow density and the manually measured PVD_by OCTA_ and PVD_by IDF_. However, this is likely to be explained by the fact that different assumptions for the calculation of flow densities and the PVD_by OCTA_ or PVD_by IDF_ were used. The automatic calculation of flow density_WF_ and flow density_Central_ principally included vessels of all diameters and the area covered by these vessels (see methods). In contrast, the manual determination of PVD_by OCTA_ and PVD_by IDF_ focused on the length of microvessels (<20 μm) in an area of interest. However, in this context it is noteworthy that in a recent study we show that automatically calculated retinal flow density showed *concordant changes* with conjunctival measured PVD_by IDF_ in hemorrhagic shock and resuscitation in sheep^25^. Further studies are needed to investigate the relationship between automatically calculated flow density and manually measured parameters of microcirculatory analysis in critical illness and during interventions in patients.

In this context, the automatically calculated flow density as a surrogate of diffusion capacity may be an important step towards establishing the evaluation of microcirculation in clinical routine and potentially enabling microcirculatory guided therapy. For example, hypovolemia as an expression of distributive shock following inflammation and sepsis frequently occurs in critically ill patients and has detrimental effects on organ perfusion and function. In this situation, the primary aim of fluid therapy is the restoration of tissue perfusion. However, targeting systemic hemodynamics (for example cardiac index or blood pressure) in the condition of distributive shock is not always indicative of adequate tissue perfusion (as initially mentioned, *loss of hemodynamic coherence*^7^), and it is well known that fluid overload, leading to edema with reduced diffusion capacity, increases mortality in critically ill patients^36–39^. In this context, surrogates of microcirculatory diffusion capacity may help to avoid hyper- and also hypovolemia^14^.

Some limitations must be considered when interpreting this study. First, the study used a commercially available OCTA device and an adaptor lens. Imaging of the sublingual microcirculation was possible, because healthy subjects were able to sit in front of the device and the lens was aligned to the sublingual region (Additional File 1, Figure A1,A2). For its use in critically ill patients (where abnormalities of the microcirculation are commonly present), the OCTA device would have to be modified. In this context, a OCTA device has been introduced, which enables imaging in recumbent patients (Flex-Modul for SPECTRALIS^®^, Heidelberg Engineering GmbH, Heidelberg, Germany)^25^. In addition, hand-held OCTA devices have been presented^40,41^.

Second, the current pilot study was able to demonstrate the basic feasibility of contactless evaluation of the sublingual microcirculation and present OCTA-derived parameters as surrogates for microcirculatory diffusion capacity. Nevertheless, to describe the functional state of the microcirculation, it would be essential to determine the blood flow velocity in OCTA images of the sublingual microcirculation (as a measure of convective flow)^26^, which was not possible with the device used. However, recent experimental and clinical studies describe an automatic measurement of blood flow velocity in OCTA images of the retina^42–44^. Third, in OCTA images of the retina and choroid, single layers are automatically identified and flow density for the respective layer calculated. To determine flow density and the PVD_by OCTA_ in the current study, the vessel leading layers of interest needed to be manually adjusted. In addition, determination of the PVD_by OCTA_ was performed semi-manually as in the analysis of IDF videos. For clinical application, automatic segmentation of vascular layers and automatic determination of vessel density would be needed, as is already possible for OCTA imaging of the retina^45,46^.

Finally, this study investigated the sublingual microcirculation in a small group of healthy subjects and each subject was evaluated only once in resting conditions. Hence the capability of OCTA to detect microcirculatory changes has not been assessed. Further research will be needed to establish whether previous findings obtained by SDF imaging and IDF illumination in critically ill patients with microvascular abnormalities can be transferred to OCTA-derived measures of the sublingual microcirculation. Due to the rapid development currently taking place in the area of OCTA technology, it is reasonable to expect these technical limitations to be overcome in the near future.

## Conclusions

The current study shows for the first time the suitability of OCTA for the evaluation of sublingual microcirculation in healthy volunteers. Contactless imaging of the sublingual microcirculation of sufficient quality was achievable with OCTA. Comparison of the perfused vessel density based on OCTA images and IDF illumination videos as surrogates of diffusive capacity showed a plausible agreement. This makes OCTA a promising tool for contactless *in vivo* evaluation of the sublingual microcirculation in critical illness. However, this study identified some technical limitations for bedside imaging of sublingual microcirculation, which need to be overcome before OCTA can be used for bedside analysis of the sublingual microcirculation in critically ill patients.

## Methods

### Ethical approval and study participants

The study was performed in accordance with the Declaration of Helsinki and was approved by local ethics committee of the Medical Chamber Westphalia-Lippe and the Westphalian Wilhelm University of Muenster, Muenster, Germany (2016–073-f-S). Voluntary study participants were recruited from our research department. To participate in the current study, patients declared their *informed consent* after they had been informed about the aims of the study, the examination procedure, potential benefits, and risks.

### Study protocol

The study was conducted as a prospective, observational study without intervention and examinations took place from November 2018 to January 2019. Imaging of the sublingual microcirculation with both OCTA and IDF illumination was performed while participants were physically resting. First, contactless measurement of the sublingual microcirculation was performed with OCTA as described below. Afterwards, videos of the sublingual microcirculation were recorded with IDF illumination. In addition, demographic data were noted, and systemic hemodynamics (blood pressure and heart rate) and peripheral oxygen saturation measured non-invasively. All images of the sublingual microcirculation (with OCTA and IDF) were recorded by the same experienced operator under the same standardized mesopic lighting conditions in the same location.

### Optical coherence tomography angiography of the sublingual microcirculation

OCTA technology has been described in detail previously^45,47,48^. Briefly, optical coherence tomography (OCT) scans of a certain region of the sublingual mucosa were performed repeatedly and OCT images were then evaluated for changes. Static tissue shows little or no change, whereas blood flow in the capillaries and larger vessels will produce differences between consecutive scans^49^. Split-spectrum amplitude-decorrelation angiography (SSADA) algorithm was used to facilitate extraction of information from the OCT angiography^50^.

Imaging of the sublingual microcirculation was conducted with a commercially available spectral domain OCT-system (AngioVue, RTVue XR Avanti SD-OCT, Optovue, Fremont, CA, USA; Additional File 1, Figure A1). The OCT device had an A-scan rate of 70,000 scans per second and used a light source centered at 840 nm (bandwidth of 50 nm). A cross-sectional image was obtained by collecting 340 adjacent punctual A-scans along a transverse coordinate, (B-scan; Fig. 1A). An OCT angiography footage of the sublingual microcirculation was composed from two times 340 B-scans, which were orthogonal to each other to correct for motion artifacts^51,52^. This resulted in a 3 × 3 mm² image (*en face* image, Fig. 1,B,C) of the sublingual microcirculation with an axial resolution of approximately 5 µm, a transversal resolution of approximately 15 µm and an acquisition time of around 3 seconds per image.

For imaging of the sublingual region, a cornea‐anterior module long adaptor lens (Optovue CAM-L S/N 43115; Optovue Inc, Fremont, California, USA) was placed in front of the imaging unit of the OCT device to enable non-contact measurements of the sublingual region (Additional File 1, Figure A2). For measurements study participants were asked to sit in front of the OCT device. The adaptor lens was aligned to the sublingual region by changing the participant’s head position (e.g., by padding with towels). Participants were allowed to maneuver the lens casing with their teeth or lips.

Only OCTA images of good quality and a quality index ≥6 were included in the analysis. Analyses of OCTA images were performed with proprietary software (ReVue 2017.1.0.151; Optovue, Fremont, CA, USA). To apply the standard quantification scheme for the macular region to the sublingual region, the sublingual vascular leading layer was manually selected in B-scans (Fig. 1A). Afterwards the flow density was automatically calculated by extracting a binary image of the vessels from the gray-scale *en face* OCTA image, and then computing the percentage of pixels of vessels in the area of interest^45^. In particular, the flow density of the central ring of the OCT angiogram (Flow density_Central_; circle 2 in Fig. 1C) and the flow density whole en face (Flow density_WF_; the average flow density of circles 1 and 2 in Fig. 1C) were calculated. At least three scans of the sublingual region at different positions were performed for each subject, and results of the examinations were noted as mean value per subject.

### Incident dark field illumination of the sublingual microcirculation

Sublingual microcirculation was measured with an IDF video microscope (CytoCam™, Braedius Medical BV, Huizen, the Netherlands). At least 5 videos of different regions of the sublingual microcirculation, each 5 s in length, were recorded for each participant. Videos of the sublingual microcirculation were reviewed using the microcirculatory image quality score (MIQS)^53^ and discarded if necessary. Analysis was conducted online using dedicated software^54^ (Capillary Mapper 1.4, University of Muenster Medical Centre, Muenster, Germany) according to the consensus conference criteria for analysis of the microcirculation in microvessels (<20 μm) following established protocols^26,55,56^. From 3–5 high quality videos of the sublingual microcirculation were analyzed in a blinded manner for each participant, noting values of microvascular flow index (MFI_by quadrants_), total vessel density (TVD), perfused vessel density (PVD_by IDF_) and proportion of perfused vessels (PPV).

### Comparison of the perfused vessel density in OCTA images and IDF videos

The segmentation of OCTA B-scans of the sublingual region were manually adjusted to include a vascular network, which was as single-layered as possible and located directly below the stratified squamous epithelium. This was done to establish whether perfused vessel density can be measured in OCTA images as effectively as in videos recorded by IDF illumination (Fig. 1A). Based on this selection, an *en face* OCTA image of the sublingual microcirculation was recalculated, which resembled a still image of an IDF illumination video of the sublingual microcirculation (Fig. 3). To compare the perfused vessel density in OCTA images (PVD_by OCTA_) with the PVD_by IDF_, image sections corresponding to the size of analyzed IDF videos (858 × 688 μm) were exported from *en face* OCTA images (box with dashed line; Fig. 3). Afterwards the vessel length relative to the image size was determined in each of the exported image sections using dedicated software (Capillary Mapper 1.4, University of Muenster Medical Centre, Muenster, Germany)^54^.

### Statistical analysis

Statistical analyses were performed using IBM SPSS^®^ Statistics 25 for Windows (IBM Corporation, Somers, New York, United States). Data are presented as median with interquartile range. Non-parametric tests were used due to the small sample size. Comparison between PVDs (by IDF and OCTA) was made using the Wilcoxon signed-rank test. Agreement between methods was analyzed following the suggestions of Bland and Altman, and a Bland-Altman-plot was drawn^27^. The Bland-Altman plot was constructed by plotting the mean difference of two values (PVD_by IDF_ and PVD_by OCTA_) for each subject against the average of those two values. The mean bias (95% confidence interval) was calculated as well as the limits of agreement (LOA) as 1.96-fold of the standard deviation of the mean bias. In addition, agreement was analyzed taking proportional bias into account. The degree of correlation between two variables was expressed as Spearman’s rank correlation coefficient ρ. The global statistical significance level was set to 0.05. Inferential statistics are intended to be exploratory (i.e. forming a basis for hypotheses), rather than confirmatory, and are interpreted accordingly.

## Supplementary information

Supplementary information

## Data Availability

The datasets used and/or analyzed during the current study are available from the corresponding author on reasonable request.
