# Author Correction: Optical coherence tomography angiography as a novel approach to contactless evaluation of sublingual microcirculation: A proof of principle study

**DOI:** 10.1038/s41598-020-68556-4

**Published:** 2020-07-07

**Authors:** Michael Hessler, Pieter Nelis, Christian Ertmer, Maged Alnawaiseh, Florian Lehmann, Christina Schmidt, Tim-Gerald Kampmeier, Sebastian Willy Rehberg, Philip-Helge Arnemann, Alexandros Rovas

**Affiliations:** 1 Department of Anesthesiology, Intensive Care, and Pain Therapy, University Hospital Muenster, Albert-Schweitzer-Campus 1, Building A1, Muenster, Germany; 2 Department of Ophthalmology, University Hospital Muenster, Domagkstraße 15, Muenster, Germany; 3 Department of Ophthalmology, University of Brussels (VUB), Laarbeeklaan 101, Jette, Belgium; 4 Department of Anesthesiology, Intensive Care, Emergency Medicine, Transfusion Medicine and Pain Therapy, Protestant Hospital of the Bethel Foundation, Burgsteig, Bielefeld, Germany; 5 Department of Medicine D, Division of General Internal Medicine, Nephrology, and Rheumatology, University Hospital Muenster, Albert-Schweitzer-Campus 1, Muenster, Germany

Correction to: *Scientific Reports* 10.1038/s41598-020-62128-2, published online 25 March 2020

The Acknowledgements section in this Article is incomplete.

“We thank Mrs. Frances Wharton for proofreading and linguistic revision of the manuscript. This study was supported by a scholarship of the German Interdisciplinary Association of Intensive Care and Emergency Medicine (Deutsche Interdisziplinäre Vereinigung für Intensiv- und Notfallmedizin; DIVI).”

should read:

“We thank Mrs. Frances Wharton for proofreading and linguistic revision of the manuscript. This study was supported by a scholarship of the German Interdisciplinary Association of Intensive Care and Emergency Medicine (Deutsche Interdisziplinäre Vereinigung für Intensiv- und Notfallmedizin; DIVI). We acknowledge support from the Open Access Publication Fund of the University of Muenster.”

