# Supplementary material for: Optical coherence tomography angiography as a novel approach to contactless evaluation of sublingual microcirculation: A proof of principle study
Source: Sci Rep. 2020 Mar 25;10:5408. doi: 10.1038/s41598-020-62128-2 (PMC7096522; doi:10.1038/s41598-020-62128-2)

Optical coherence tomography angiography as a novel approach to contactless evaluation of sublingual microcirculation: A proof of principle study

Michael Hessler[1#], michael.hessler@uni-muenster.de;

Pieter Nelis[2,3#], pieter.nelis@ukmuenster.de;

Christian Ertmer[1*], ertmer@anit.uni-muenster.de;

Maged Alnawaiseh[2], maged.alnawaiseh@ukmuenster.de;

Florian Lehmann[1], florian.lehmann@uni-muenster.de;

Christina Schmidt[1], christina.schmidt@ukmuenster.de;

Tim-Gerald Kampmeier[1], tim.kampmeier@gmail.com;

Sebastian Willy Rehberg[4], sebastianwillyrehberg@gmail.com;

Philip-Helge Arnemann[1#], arnemann@anit.uni-muenster.de;

Alexandros Rovas[5#], alexandros.rovas@ukmuenster.de.

Additional File 1

Figure A1 Optovue XR Avanti with AngioVue

Legend: The optical coherence tomograph used in the study, built by Optovue Inc., Fremont, California, USA.

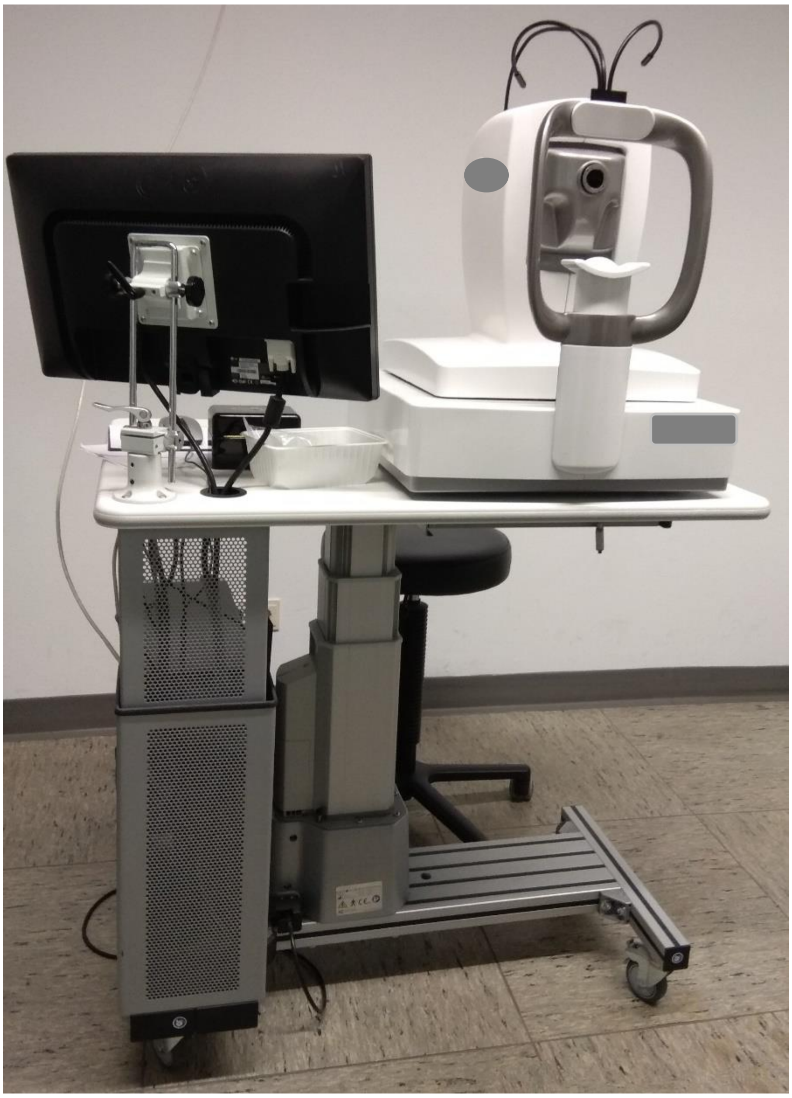

Figure A2 Cornea-anterior module long adaptor lens

Legend: The adaptor lens was placed in front of the imaging unit of the optical coherence tomograph for contactless imaging of the sublingual region enabling a 6x6mm field of view (Lens: Optovue CAM-L S/N 43115; Optovue Inc, Fremont, California, USA).

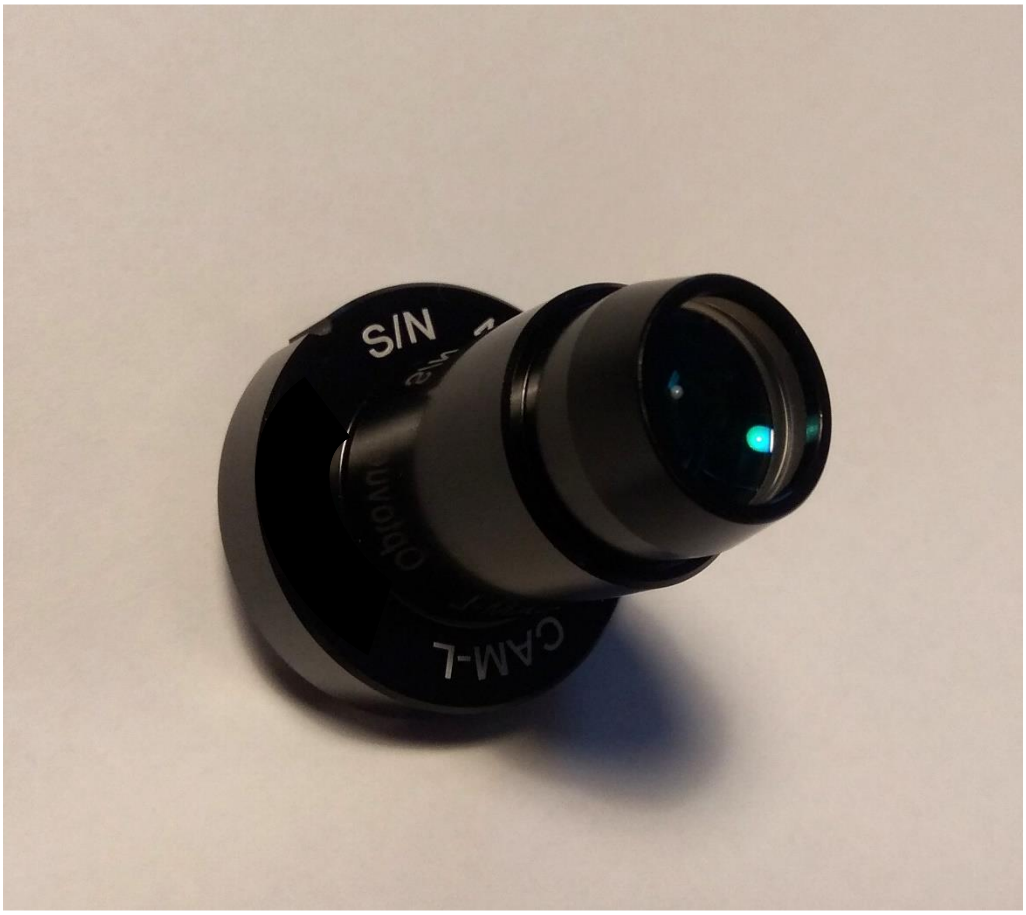

Figure A3 **Boxplots of the perfused vessel density of the sublingual microcirculation based on incident dark field illumination and optical coherence tomography angiography.**

Abbreviations: IDF, incident dark field illumination, OCTA, optical coherence tomography angiography; PVD, perfused vessel density.

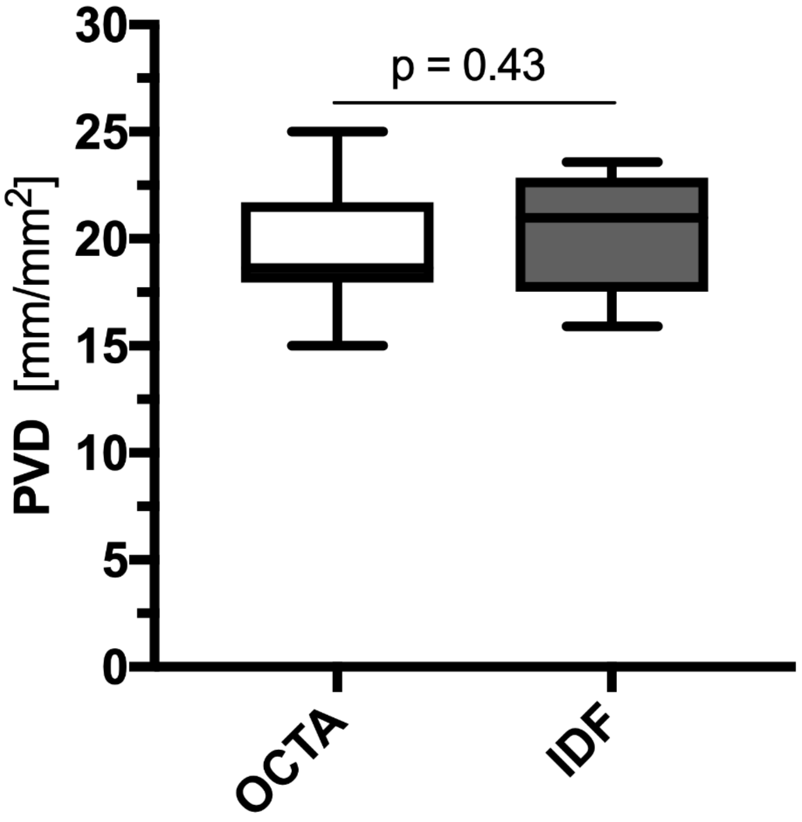

Supplement: Supplementary file 1 — Supplementary information [file 41598_2020_62128_MOESM1_ESM.pdf]
